# Commissioning of a motion system to investigate dosimetric consequences due to variability of respiratory waveforms

**DOI:** 10.1120/jacmp.v17i1.5921

**Published:** 2016-01-08

**Authors:** Ashley J. Cetnar, Joshua James, Brain Wang

**Affiliations:** ^1^ Department of Radiation Oncology University of Louisville School of Medicine Louisville KY USA

**Keywords:** motion management, HexaMotion, respiratory gating, ITV

## Abstract

A commercially available six‐dimensional (6D) motion system was assessed for accuracy and clinical use in our department. Positional accuracy and respiratory waveform reproducibility were evaluated for the motion system. The system was then used to investigate the dosimetric consequences of respiratory waveform variation when an internal target volume (ITV) approach is used for motion management. The maximum deviations are 0.3 mm and 0.22° for translation and rotation accuracy, respectively, for the tested clinical ranges. The origin reproducibility is less than ±0.1 mm. The average differences are less than 0.1 mm with a maximum standard deviation of 0.8 mm between waveforms of actual patients and replication of those waveforms by HexaMotion for three breath‐hold and one free‐breathing waveform. A modified gamma analysis shows greater than 98% agreement with a 0.5 mm and 100 ms threshold. The motion system was used to investigate respiratory waveform variation and showed that, as the amplitude of the treatment waveform increases above that of the simulation waveform, the periphery of the target volume receives less dose than expected. However, by using gating limits to terminate the beam outside of the simulation amplitude, the results are as expected dosimetrically. Specifically, the average dose difference in the periphery between treating with the simulation waveform and the larger amplitude waveform could be up to 12% less without gating limits, but only differed 2% or less with the gating limits in place. The general functionality of the system performs within the manufacturer's specifications and can accurately replicate patient specific waveforms. When an ITV approach is used for motion management, we found the use of gating limits that coincide with the amplitude of the patient waveform at simulation helpful to prevent the potential underdosing of the target due to changes in patient respiration.

PACS numbers: 87.55.Kh, 87.55.Qr, 87.56.Fc

## I. INTRODUCTION

It is challenging to provide quality assurance for managing respiratory motion in radiation oncology. Motion from respiration is complex and patient‐specific. Even for the same patient, breathing can vary between simulation and treatment, or among treatment fractions.^(1)^ There are several ways to account for motion in the treatment process. Respiratory gating can be used to treat only when the tumor is in a certain phase, minimizing dose to surrounding tissue. However, treatment time is increased with the gating approach. More importantly, gating relies on a correlation between the tumor and an external surrogate, which is difficult to validate.^2^, ^3^, ^4^

An internal target volume (ITV) approach uses the extent of the target motion to create a margin that encompasses the tumor over all phases of respiration, but this technique treats more healthy tissue around the target.^5^, ^6^, ^7^ Whichever method is used to account for respiratory motion must be thoroughly tested to ensure proper treatment of the patient.

AAPM Task Group Report 142 provides quality assurance (QA) recommendations for respiratory management.^(8)^ All of the systems require synchronization of the treatment beam with the respiratory motion. Specific tests include the temporal accuracy and the calibration of a surrogate for respiratory phase and amplitude, and TG‐142 recommends the use of a dynamic phantom that can simulate human organ motions to test the target localization and gated treatment accuracy. Several motion phantoms are available commercially and others have been designed in‐house for research at some institutions.^9^, ^10^, ^11^ However, most of these phantoms are limited either by the inability to reproduce a patient specific waveform or in the degrees of freedom of the motion platform.

The HexaMotion system (ScandiDos, Uppsala, Sweden) can simulate patient‐specific motion in six dimensional space. The motion platform works in conjunction with the Delta^4^ phantom (ScandiDos) to verify dose to a moving target. This motion system has the ability to dynamically simulate human respiratory motion and verify both the phase and amplitude of the waveform. By calibrating the diodes with an ion chamber, the dose output can be verified. While the HexaMotion device has been used to investigate dosimetric effects of dynamic multileaf collimator (DMLC) tracking, it has not been systematically characterized in the literature.^(12)^ In this study, we commissioned the general functionality of the HexaMotion system in order to evaluate the dosimetric consequences of respiratory waveform variation.

Some studies have shown that dosimetric uncertainties introduced by irregular breathing patterns tend to average out over the course of several weeks of intensity‐modulated radiation therapy (IMRT) treatments.^(13)^ However, such uncertainties are less forgiving for stereotactic body radiation therapy (SBRT) treatments with tight margins and a small number of fractions. For example, one such study evaluated the dosimetric effects of respiratory motion and found that the pattern of change was independent of respiratory amplitude.^(11)^ That study only provided evaluation for conventional fractionation treatments, not for SBRT applications. Other limitations include that the motion was only in one dimension and the results were collected using Gafchromic film which requires additional time for scanning and analysis. Our study is specific to SBRT treatments using an ITV technique for motion management.

## II. MATERIALS AND METHODS

**Figure 1:**
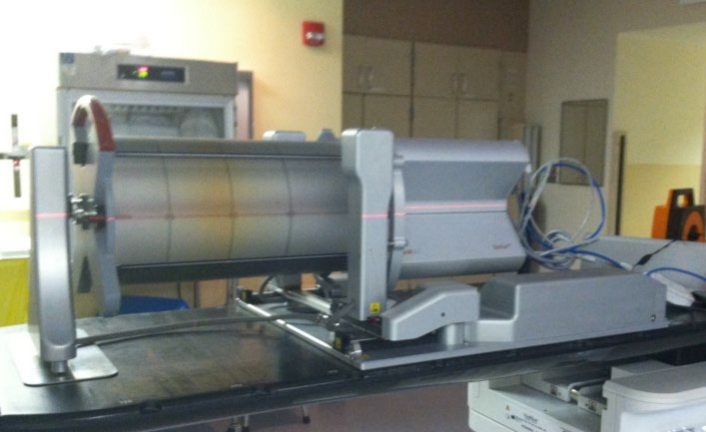
HexaMotion system including the Delta^4^ phantom and the motion platform.

The HexaMotion system, shown in Fig. 1, uses the Delta^4^ phantom with HexaMotion base to replicate 6D motion from patients. The Delta^4^ phantom contains 1,069 p‐type diodes in two orthogonal planes which are surrounded by a cylindrical phantom. Delta^4^ has been commissioned extensively in the literature showing the phantom to be both capable of precise and accurate dose measurement and efficient for measuring IMRT QA.^14^, ^15^, ^16^, ^17^

### A. Commissioning of HexaMotion

#### A.1 Translation accuracy

The translational motion of the phantom was validated using the optical camera system from the Varian TrueBeam Respiratory Motion Management system (Varian Medical Systems, Palo Alto, CA) with an uncertainty of 0.25 mm. The reflective block from the Varian system was placed on top of the Delta^4^ phantom and tracked in service mode using the optical camera calibration. The HexaMotion software was used to move the phantom in each direction independently (lateral, longitudinal, and vertical), and the displacement was recorded using the coordinates from the reflective block. The phantom was also moved with components in all three dimensions simultaneously to record the combined motion.

#### A.2 Rotation accuracy

The rotational motion of the phantom was validated using a two‐axis graphic inclinometer (Level Developments Ltd, Surrey, UK) with an uncertainty of $\pm 0.05^{\circ }$. This digital level was secured on the top of the phantom centered on the middle of the diode planes. The HexaMotion software was used to move the phantom independently in pitch and roll, and the corresponding angle was measured.

#### A.3 Origin reproducibility

The reproducibility of the origin was tested using the same experimental technique as the translation accuracy by tracking the reflective block using the optical camera calibration in TrueBeam service mode. The initial absolute position was recorded and the phantom was moved to the maximum range in each direction. The phantom was then returned to the origin and the new position was recorded. To measure the reproducibility within the session, eight measurements were recorded (n=8). This experiment was repeated in three different sessions to test the reproducibility from session to session.

#### A.4 Waveform reproducibility

Several patient specific waveforms were collected using the Varian Real‐Time Position Management (RPM) system. The system was only used to aid in the generation of a waveform representative of a realistic patient waveform and was not used for measurement. These waveforms were transferred to the HexaMotion system, and these HexaMotion files were used as a baseline representing the patient waveform. The motion of the phantom was then recorded using the TrueBeam Respiratory Motion Management system. The recorded waveform of the HexaMotion from the TrueBeam system was compared to the baseline HexaMotion waveform by translating the data in time and position to minimize the difference between the two waveforms. The difference and standard deviation were recorded in all three dimensions of motion for various clinically relevant waveforms.

An additional analysis was performed that compared the waveforms to defined tolerances similar to a gamma index.^(18)^ In this analysis, distance and time were used instead of dose and distance used in the traditional gamma analysis, as shown in (1), (2). Since the largest component of motion was in the A/P direction, this was the direction considered for the analysis.

(1) $$\Gamma =\sqrt{{\left(\frac{x_{o}-x_{i}}{\Delta X}\right)}^{2}+{\left(\frac{t_{o}-t_{i}}{\Delta T}\right)}^{2}}$$

(2) γ=MIN(Γ)

where $x_{o}$ represents a point from the reference waveform, $x_{i}$ represents a point from the HexaMotion waveform, and ΔX is the criterion for agreement. Similarly, $t_{o}$ represents the reference time point, $t_{i}$ is the point from the HexaMotion waveform, and ΔT is the time criterion. A gamma value less or equal to one represents a matching point for the given tolerances.

### B. Dosimetric consequences of respiratory variation

Dosimetric consequences of waveform variation were investigated using an ITV approach for planning. Studies have shown that the range of tumor motion is complex and can range from 0–24 mm in the AP direction for lung tumors.^(19)^ For this reason, we chose a waveform with an amplitude of 18 mm to be used by HexaMotion to simulate respiratory motion during the 4D CT of the Delta^4^ phantom. A cylindrical target volume was created during planning to cover the inner diode region of the Delta^4^ for each of the 10 phases of respiratory motion. The cylinder had a diameter of 6.7 cm and height of 6.7 cm centered on the inner diode region (6×6 cm) of the Delta^4^ to encompass all of the inner diodes. An identical cylinder was created on each of the phases. The ITV was made by superimposing the cylindrical volumes from all 10 phases into one structure. A VMAT plan was optimized for delivery.

**Figure 2:**
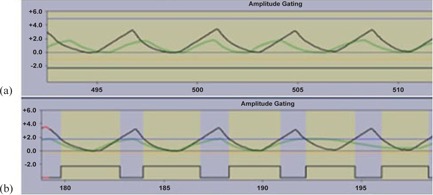
Example of waveforms for two gating limits: open limits to allow uninterrupted treatment (a) and closed limits to interrupt beam if treatment amplitude is greater than simulation amplitude (b). Horizontal lines represent the upper (blue) and lower (orange) limits. The black curve is an example of a respiratory waveform during treatment greater than the original, and the green curve is the waveform from CT simulation. The yellow shaded area indicates that the beam is on.

The plan was first delivered on the Varian TrueBeam using the same waveform from simulation shown by the green reference waveform in Fig. 2(a). The treatment was repeated using four different waveforms with increasing amplitude from 5‐20 mm greater than the original amplitude in the anterior posterior direction. One example is shown by the black curve in Fig. 2(a). The delivery was again repeated with the higher amplitude waveforms, but gating limits were used to terminate the beam during delivery if the amplitude of the treatment waveform became greater than the amplitude of the original waveform from simulation, as shown in Fig. 2(b).

## III. RESULTS

### A. Commissioning of HexaMotion

#### A.1 Translation accuracy

The displacement of the HexaMotion was recorded using the coordinates from the reflective block affixed to the motion system. The vendor reports an accuracy of 0.25 mm for the system. Each of the axes (lateral, longitudinal, and vertical) was moved independently and each of the coordinates were recorded at each position. The combined motion of moving the coordinates simultaneously was also recorded. The differences between the expected and measured translational position in each direction are shown in Table 1.

**Figure 3:**
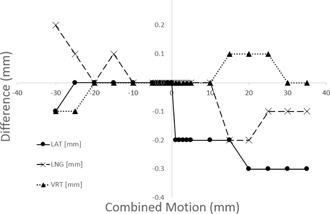
Example of translation difference for HexaMotion with movement in each of the directions (lateral, longitudinal, and vertical).

Figure 3 shows an example of the data from the combined motion. For example, the data shown at −30 mm is the recorded difference when lateral, longitudinal, and vertical positions all are moved to −30 mm.

**Table 1:** The first three rows show data from when each of the axes was moved independently, and the last is the combined motion with each of the components together.

		*Average (mm)*	*SD (mm)*	*Max. Difference (mm)*
LAT Alone	LAT	0.0	0.1	0.2
	LNG	0.0	0.2	0.3
	VRT	‐0.1	0.1	0.2
LNG Alone	LAT	0.0	0.1	0.1
	LNG	0.0	0.0	0.1
	VRT	0.0	0.1	0.1
VRT Alone	LAT	0.0	0.0	0.1
	LNG	0.0	0.0	0.1
	VRT	0.0	0.0	0.1
Combined Motion	LAT	‐0.1	0.1	0.3
	LNG	0.0	0.1	0.2
	VRT	0.0	0.0	0.1

#### A.2 Rotation accuracy

**Figure 4:**
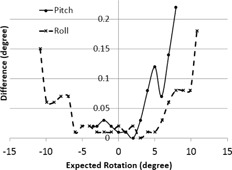
Measured difference in rotation for pitch and roll using two‐axis inclinometer.

Rotation was measured using a dual axis inclinometer with an uncertainty of $\pm 0.05^{\circ }$. The differences between the expected and measured rotation for roll and pitch are shown in Fig. 4. Please note that the measurements for pitch are not symmetric about the origin because of the physical limitation of the HexaMotion system to obtain a greater than 3° pitch in the negative direction.

#### A.3 Origin reproducibility

The reproducibility of the origin was tested by recording the initial origin position, moving the phantom to the maximum range in each direction, and returning the phantom to the origin. The origin was stable throughout the three separate sessions with eight measurements each, and the results are shown in Table 2.

**Table 2:** HexaMotion origin reproducibility.

	*Average (mm)*	*SD (mm)*	*Max. Difference (mm)*
LAT	−0.1	0.1	0.2
LNG	0.0	0.1	0.1
VRT	0.0	0.1	0.1

#### A.4 Waveform reproducibility

The baseline HexaMotion waveforms were compared to the TrueBeam motion of the HexaMotion system. The difference in position was calculated point by point between the original and measured HexaMotion waveforms in each of the translational dimensions. Table 3 shows the average of the difference and standard deviation (SD) for four (n=4) different waveforms composed of three breath‐hold waveforms and one free‐breathing waveform. The amplitudes of the breath‐hold portion of the waveforms are approximately 1.0 cm and 1.5 cm above normal respiration, and the third breath‐hold waveform is defined at full expiration. The amplitude of the normal free‐breathing waveform was 1.7 cm.

**Figure 5:**
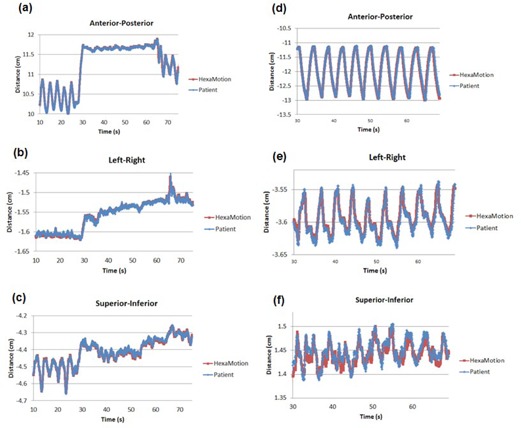
Comparison of the original patient baseline waveform with the reproduced waveform from HexaMotion measured in the anterior–posterior, right–left, and superior–inferior directions for a breath‐hold waveform ((a)‐(c)) and free‐breathing waveform ((d)‐(f)). Please notice the different scales for the y‐axes to maximize the use of the plot area.

An example of the results from free breathing and breath hold are shown in Fig. 5. The red lines representing the HexaMotion motion are overlaid with the blue lines of the baseline waveform. A typical representation of a breath‐hold waveform is shown in the left column (Fig. 5(a) to (c)), which corresponds to the Breath‐hold 1 data in Table 3. A free‐breathing waveform is shown in the right column (Fig. 5(d) to (f)), which corresponds to the Free‐breathing waveform in Table 3.

The average difference is less than the manufacturer's specifications of 0.5 mm for all waveforms, in all directions. However, the greatest deviation was in the free‐breathing waveform in the anterior/posterior direction with a standard deviation of 0.8 mm, as shown in Table 3. We further investigated the differences which occurred in the high‐gradient regions. Because of this, an additional analysis was created to better represent the reproducibility of the system. Each of the waveforms was evaluated using the modified gamma analysis for tolerances of 1 mm, 0.5 mm, and 0.1 mm with a time tolerance of 100 ms.^(8)^ The modified gamma pass rates are shown in Table 4.

**Table 3:** Average difference and SD between waveforms of actual patients and replication of waveforms by HexaMotion for three breath‐hold and one free‐breathing waveform.

	*Anterior/Posterior*		*Right/Left*		*Superior/Inferior*	
	*Average (mm)*	*SD (mm)*	*Average (mm)*	*SD (mm)*	*Average (mm)*	*SD (mm)*
Breath‐hold 1	0.0	0.3	0.00	0.04	0.05	0.09
Breath‐hold 2	0.0	0.3	0.00	0.07	0.00	0.09
Breath‐hold 3	0.0	0.4	0.00	0.04	0.0	0.1
Free‐breathing	0.0	0.8	0.00	0.07	0.0	0.1

**Table 4:** Modified gamma pass rates comparing waveforms using a 100 ms tolerance for time and 1.0, 0.5, and 0.1 mm tolerances for distance.

	*1 mm*	*0.5 mm*	*0.1 mm*
Breath‐hold 1	100%	99.6%	85.9%
Breath‐hold 2	99.7%	99.4%	92.0%
Breath‐hold 3	100%	100%	99.9%
Free‐breathing	100%	98.2%	81.2%

### B. Dosimetric consequences of respiratory variation

**Figure 6:**
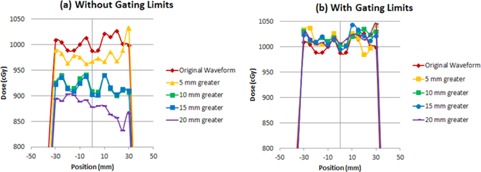
Dosimetric results of varying the amplitude from the original waveform without gating limits (a) and with gating limits (b).

The amplitude of the patient's breathing at treatment can affect the delivered dose to the target when an ITV approach is used for motion management. The PTV covered by the test plan was cylindrical, with a radius of 3.35 cm to encompass the inner diode region of the Delta^4^ which extends 3 cm from the center. Since the configuration of the diode planes was perpendicular in an “X” configuration, we analyzed the measured dose for the rows of diodes on the anterior portion of the ITV at 3 cm. Figure 6 shows the average of the measured dose profiles through the diodes along the superior/inferior periphery of the target volume for treatment delivery using the original waveform and the higher amplitude waveforms, with and without gating limits.

Table 4 shows that as the amplitude of the treatment waveform increases above that of the original waveform from simulation, the diodes representing the periphery of the target are receiving less dose than expected. With the largest amplitude of 20 mm, the average dose to the diodes on the edge of the target is 12% lower than expected, as shown in Table 5. However, by using gating limits to terminate the beam outside of the original amplitude, the results are similar dosimetrically with an average percent difference within 2%.

**Table 5:** Average percent difference from the dose collected with the original waveform from simulation.

	*Without Gating Limits*		*With Gating Limits*	
*Amplitude*	*Average Difference*	*Max. Difference*	*Average Difference*	*Max. Difference*
5 mm greater	−2%	−6%	1%	4%
10 mm greater	−8%	−13%	2%	3%
15 mm greater	−9%	−12%	1%	3%
20 mm greater	−12%	−17%	1%	5%

## IV. DISCUSSION

All of the translational measurements for the HexaMotion system deviated less than 0.3 mm, which are within the manufacturer's specification of 0.5 mm. The difference from the expected value increases as the phantom is moved farther from the origin. The manufacturer specifies a rotational accuracy of 0.2°, and our measurements are consistent with this claim within the uncertainty of our measuring device. Similar to translation, the deviation from the expected position increases as it rotates farther from the origin. Therefore, we recommend that the system be set up manually to machine isocenter, using the software for fine adjustments only.

The system was able to accurately reproduce realistic patient waveforms based on the results of our waveform analysis. The data were compared by translating the data temporally and adjusting the absolute position of each of the dimensions to minimize the difference in the waveforms. This adjustment was made to analyze the data because the original waveform was acquired in the RPM coordinate system and the comparison waveform was acquired in the TrueBeam coordinate system. Differences in the orientation and origin of the two coordinate systems resulted in a difference in the recorded absolute position of the motion surrogate. However, the phase and amplitude of the data are preserved, which is of the most importance for this analysis. The average difference between the waveforms is 0.1 mm or less and the modified gamma pass rates are greater than 98% for 0.5 mm with 100 ms tolerance, which agree with the manufacture's specifications of 0.5 mm. A limitation of this study is that we are not able to report dynamic system rotation since the current versions of RPM and TrueBeam Motion Management system do not track or record rotation.

We have found that the HexaMotion system can be effectively integrated into an institution with existing motion management devices, including the Varian RPM and TrueBeam Respiratory Motion Management systems. In evaluating the dosimetric effect of respiratory variation from simulation to treatment delivery, an 8%–12% underdosing of the target volume, even for 1 fraction, could be clinically significant when treating a small number of fractions, such as SBRT delivery. The underdosing is caused by a potentially incorrect assumption that the patient breathes similarly during treatment delivery and 4D CT simulation. This assumption is a key factor when using the ITV approach for motion management. The target volume is constructed from imaging data acquired by the 4D CT, and the plan is optimized to cover the target volume with the prescription dose during its full extent of motion as determined from the 4D CT. At the time of treatment, if the patient breathes similarly to that during 4D CT simulation, it is reasonable to assume that the target volume stays within the prescribed isodose surface during its full extent of motion. However, the patient may breathe differently at the time of treatment, causing the tumor motion to be different than it was during the acquisition of the 4D CT scan used for treatment planning. If the breathing amplitude is smaller during treatment delivery, the extent of target motion decreases and the target coverage is not affected. However, if the breathing amplitude is larger during treatment delivery, the extent of target motion increases, causing the target to move outside of the prescription isodose surface optimized during the planning process. The effect could be even more significant than shown in this study if coplanar arcs are used to deliver dose only through the axial plane due to the very steep dose gradient at the field edge in the superior/inferior direction.

Therefore, when using the ITV technique for SBRT planning, we found the use of gating limits that coincide with the amplitude of the patient waveform at 4D CT simulation helped to prevent the potential underdosing of the ITV due to changes in patient respiration. We recognize the limitation of the study's assumption that the motion of the external surrogate replicates the actual internal motion of the target. In reality, the motion of the target relative to the external surrogate is patient‐specific, and any dosimetric consequences due to a change in the waveform measured by the external surrogate would be dependent on the motion of the target relative to the surrogate. However, by implementing gating limits based on the motion of the external surrogate from simulation, the motion of the target while the treatment beam is on will be confined to the range from the 4D CT simulation. Any additional motion of the target represented by an increase in the amplitude of the respiratory waveform recorded from the external surrogate will not significantly affect the dosimetric accuracy of the treatment when gating limits are applied.

## V. CONCLUSIONS

The general functionality of the HexaMotion system performs within the manufacturer's specifications and can accurately replicate patient‐specific waveforms. It can be used to investigate clinical issues involving the management of respiratory motion. We used the device to determine the dosimetric consequences of a patient's respiratory waveform having greater amplitude at treatment compared to simulation when an ITV approach is used for motion management. We found the use of gating limits that coincide with the amplitude of the patient waveform at 4D CT simulation help to prevent the potential underdosing of the ITV due to changes in patient respiration. Future work can use this device to evaluate other motion management techniques, such as deep inspiration breath‐hold, electromagnetic tracking, and optical surface tracking.
